# Evaluation of Arterial Stiffness Using Pulse Wave Velocity and Augmentation Index in Patients with Chronic Venous Insufficiency

**DOI:** 10.1155/2018/5437678

**Published:** 2018-12-02

**Authors:** Mustafa Dogdus, Onur Akhan, Mehmet Ozyasar, Ahmet Yilmaz, Mehmet Sait Altintas

**Affiliations:** ^1^Karaman State Hospital, Department of Cardiology, 70100 Karaman, Turkey; ^2^Ege University School of Medicine, Department of Cardiology, 35100 Izmir, Turkey

## Abstract

**Background and Objectives:**

Chronic venous insufficiency (CVI) is a common pathology of the circulatory system and is associated with a high morbidity for the patients and causes high costs for the healthcare systems. Arterial stiffness has been shown to be a predictor of cardiovascular events and mortality. The relationship between CVI and arterial stiffness using pulse wave velocity (PWV) and augmentation index (Aix) was evaluated in this study.

**Methods:**

Sixty-two patients with the stage of C3-C5 chronic venous disease (CVD) and 48 healthy subjects were enrolled in the study. To assess arterial stiffness, all cases were evaluated with I.E.M. Mobil-O-Graph brand ambulatory blood pressure monitor device. PWV and Aix were used to assess arterial stiffness in this study.

**Results:**

The mean age was 61.9±11.05 years and 54 % of the patient population was females. PWV and Aix were significantly higher in CVI patients than controls (8.92±1.65 vs. 8.03±1.43, p=0.001; 25.51±8.14 vs. 20.15±9.49, p=0.003, respectively) and also positive linear correlation was observed between CVI and all measured arterial stiffness parameters (r=0.675 for CVI and PWV, r=0.659 for CVI and Aix, respectively). A PWV value of > 9.2 has 88.9 % sensitivity and 71.4 % specificity to predict the presence of CVI.

**Conclusions:**

PWV and Aix are the most commonly used, easy, reproducible, reliable methods in the clinic to assess arterial stiffness. Logistic regression analysis showed that PWV and Aix were the independent predictors of CVI. PWV has the sensitivity of 88.9 % and specificity of 71.4 % to detect the presence of CVI.

## 1. Introduction

Chronic venous disease (CVD) is a common pathology of the circulatory system that has high prevalence, high cost of diagnosis and treatment, significant loss in manpower, and adverse effects on the quality of life of the patients [1, 2]. This condition encompasses a cascade of pathophysiological consequences arising from venous hypertension in the lower extremities, which can have variable etiologies. Most commonly, this venous hypertension is associated with venous reflux due to poor functioning or incompetent venous valves, which ultimately reduces venous return, leading to blood pooling, hypoxia, and inflammation.

CVD includes the full spectrum of signs and symptoms associated with classes C0 to C6 of the Clinical–Etiology–Anatomy–Pathophysiology (CEAP) classification and Chronic Venous Insufficiency (CVI) is a severe form of the disease.

Arterial stiffness is a strong predictor of cardiovascular (CV) events and all-cause mortality [3–5]. Various physiological and pathophysiological parameters influence arterial stiffening including age, gender, blood pressure, smoking, and diseases such as hypertension, diabetes, renal failure, and hypercholesterolemia. Thus, the assessment of arterial stiffness has become a widely used tool to investigate the function of the arteries in epidemiologic and clinical studies. It has also been shown that increased arterial stiffness may predict cardiovascular events in asymptomatic individuals without overt cardiovascular disease. In most studies, increased PWV values in healthy normotensive individuals indicated subclinical vascular and cardiac organ damage [6]. Measuring arterial stiffness may, therefore, identify patients at risk at an early stage.

Traditionally, arterial stiffness has been assessed by pulse wave velocity (PWV), a noninvasive parameter which has been shown to predict cardiovascular mortality [7–10]. In addition, pulse wave analysis has been increasingly used to determine the augmentation index (Aix), a parameter that describes the effect of pulse wave reflection on the central aortic pressure configuration. PWV and Aix are the most commonly used, easy, reproducible, reliable, best prognostic methods in the clinic to assess arterial stiffness [11–13].

Venous pathology develops when venous pressure increases and blood return is insufficient. Continuous venous hypertension leads to some histological changes known as “venous hypertensive microangiopathy” in capillary networks. These changes are capillary network curl, expansion, and increase on the endothelial surface. It is inevitable that the changes in the venous system due to the continuity of vascular structure also affect the arterial system. Arterial stiffness in CVI may be affected due to the continuity of vascular structure. Although arterial stiffness is known in coronary heart diseases and arterial pathologies, there is no sufficient information about venous diseases. As a result, we aimed to evaluate the arterial stiffness in CVI patients with PWV and Aix.

## 2. Methods

### 2.1. Study Population

Sixty-two patients with the stage of C3-C5 CVD and 48 age/sex and body mass index (BMI) matched healthy subjects were enrolled into the study between November 2015 and April 2016. CVD was evaluated according to CEAP classification. Patients fulfilling CEAP class 3, 4, and 5 were included in this study. The exclusion criteria were history of myocardial infarction, percutaneous coronary intervention (PCI) and coronary bypass grafting, cardiac pacemaker, heart failure, cardiomyopathy, valvular heart diseases, arrhythmia, LVEF < 55%, thyroid dysfunction, chronic renal failure (GFR <60 ml/min/1,73 m2), peripheral artery disease ( ankle brachial index<0.9), connective tissue disorders, malignancy and use of cardiotoxic medication, uncontrolled hypertension, congenital heart disease, chronic obstructive lung disease, congenital CVD, secondary CVD, and active leg ulcers (CEAP class C6). The study was approved by the local ethics committee. All of the patients in the study signed an informed consent form.

### 2.2. Demographic, Clinical, and Echocardiographic Evaluation of Patients

Body mass index (BMI, kg/m2) was calculated by dividing body weight in kilograms by the square of body height in meters. Transthoracic echocardiography assessment (Vivid E9; GE Healthcare, Horten, Norway) was performed in patients according to the echocardiography guidelines. All images were accrued from patients in the left lateral decubitus position and connected to ECG.

Diabetes mellitus (DM) was defined as fasting plasma glucose ≥ 126mg/dl or plasma glucose level ≥ 200mg/dl 2 hours after the 75mg oral glucose tolerance test or HbA1C ≥ 6.5% or patients using antidiabetic medications. Hypertension (HT) was defined by a previous diagnosis of hypertension or the presence of SBP ≥140 mmHg or DBP ≥ 90mmHg. Hyperlipidemia (HLP) was defined as total cholesterol >190mg/dl or previous diagnosis of dyslipidemia. Cigarette smoking was defined as smoking ≥1 cigarettes a day for at least 1 year, without an attempt to quit.

### 2.3. Arterial Stiffness Measurement by Using PWV and Aix

I.E.M. Mobil-O-Graph ambulatory blood pressure monitor device, which automatically measure pulse wave velocity (PWV), central blood pressure (cBP), augmentation index (Aix), and central pulse pressure (cPP), was used to evaluate arterial stiffness. Ambulatory blood pressure measuring device recorded data from patients for 24 hours. PWV, Aix, and cBP from these parameters were used in this study.

### 2.4. Statistical Analysis

SPSS 25.0 (IBM Corporation, Armonk, New York, United States) program was used to analyze the variables. Quantitative variables were expressed as mean ± SD (standard deviation) and median range (Maximum-Minimum) according to the normal distribution of the variable, and categorical variables were expressed as numbers and percentages. The normal distribution of the data was evaluated by the Shapiro-Wilk test and the variance homogeneity was evaluated by the Levene test. The Independent-Samples T test was used with the Bootstrap results when comparing two independent groups with one according to the quantitative data, and the Mann–Whitney U test was used together with the Monte Carlo results. To compare categorical variables, Pearson Chi-Square and Fisher Exact tests were tested using exact results. The odds ratio was used to determine the most important risk factor from categorical significant risk factors. Binary logistic regression analysis and Receiver operator characteristic curves (ROCs) were used to analyze the sensitivities of PWV and Aix to predict the presence of CVI. Variables were examined at 95% confidence level (Cl) and p <0.05 was considered to indicate a statistically significant difference.

## 3. Results

The mean age was 61.9±11.05 years and 54 % of the patient population was female. Baseline characteristics of study groups were compared in Table 1. 68% of the general population had HT and 40% had DM and 62.5 % of the patients were still smoking (Table 1).

There were not any significant differences between groups for age, gender, HT, DM, HLP, LVEF, BMI, and smoking (Table 1). Also there was no significant difference between the groups in terms of central systolic and diastolic blood pressure values (p=0.262 and p=0.159, respectively) (Table 1).

TG was significantly higher in patients with CVI than controls (158.74±58.72 mg/dl vs. 122.50±45.65 mg/dl, p=0.040) (Table 1).

PWV and Aix were significantly higher in CVI patients than controls (8.92±1.65 vs. 8.03±1.43, p=0.001; 25.51±8.14 vs. 20.15±9.49, p=0.003, respectively) (Table 1) and also positive linear correlation was observed between CVI and all measured arterial stiffness parameters (r=0.675 for CVI and PWV, r=0.659 for CVI and Aix, respectively) (Table 2).

PWV (p<0.001, Odds ratio (OR) =5.36, 95% Confidence interval (C.I.) =2.35–12.26), Aix (p<0.001, OR=4.69, 95% C.I. =1.86–11.84), and TG (p=0.028, OR=2.96, 95% C.I. =1.34–6.55) were the independent predictors of CVI (Table 3).

**Figure 1:**
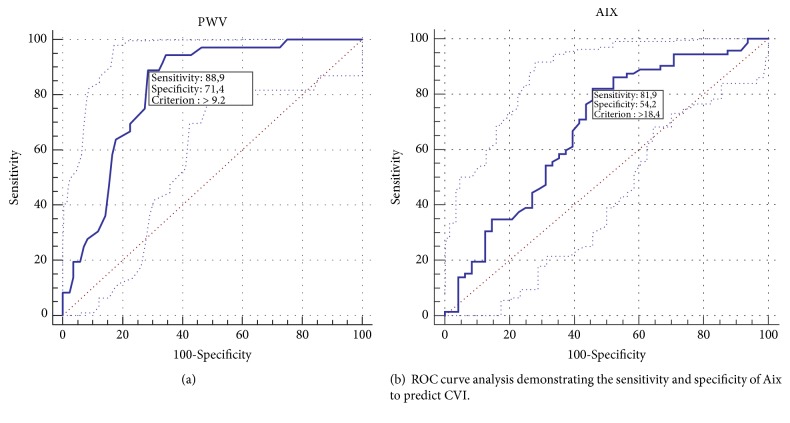


ROC analyses were performed to find out ideal arterial stiffness cut off values to predict the presence of CVI. A PWV value of > 9.2 has 88.9 % sensitivity and 71.4 % specificity and an Aix value of > 18.4 has 81.9 % sensitivity and 54.2 % specificity to detect the presence of CVI (AUC 0.826 (p<0.001) and AUC 0.795 (p<0.001), respectively) (Figures 1(a), 1(b) and Table 4).

## 4. Discussion

CVD is a progressive medical condition that results from venous hypertension. It is already a major and growing global medical problem, with a high economic burden. Chronic inflammation and “venous hypertensive microangiopathy” are the principal basis behind the pathophysiological mechanisms that potentiate disease progression and produce the signs and symptoms of CVD. Brought on by chronic venous hypertension, the accumulating leukocytes and damaged venous endothelium initiate inflammatory cascades in the distended veins, which then propagate into the microvasculature and surrounding tissues. This chronic inflammation further degrades venous integrity and function, resulting in diminished venous return, fluid accumulation, tissue fibrosis, atrophy, and ulceration in severe cases [14].

An injury or dysfunction in a part of vascular tree may affect all vascular system. Atherosclerosis, arteriolosclerosis, and CVD share common risk factors and mediators. Therefore, CVD may be an accepted form of vascular sclerosis and vascular system should be evaluated in continuum. Arterial stiffness in CVI may be affected due to this continuity of vascular structure. Therefore, arterial stiffness using PWV and Aix in CVI patients was investigated in this study. The present study showed that PWV and Aix were significantly increased in patients with CVI compared to controls. Logistic regression analysis showed that PWV, Aix, and TG were the independent predictors of CVI.

Ozpelit et al. reported that among the arterial stiffness parameters, central aortic pressure, Aix, and PWV were slightly higher in patient with CVD [15]. Similarly, we found that PWV and Aix were significantly increased in patients with CVD.

Aykan AC et al. showed that cardioankle vascular index (CAVI) is independently increased in CVI patients and a CAVI value of > 7.9 had a sensitivity of 64.4% and a specificity of 94.7% for predicting the presence of CVI [16]. However, the present study showed that a PWV value of > 9.2 has 88.9 % sensitivity and 71.4 % specificity and an Aix value of > 18.4 has 81.9 % sensitivity and 54.2 % specificity to detect the presence of CVI. One of the reasons for these differences of sensitivity and specificity may be using different parameters to assess arterial stiffness. The present study was found to be more sensitive for predicting CVI. Also, in their study there was not enough information about other physiological and pathophysiological variables that may affect arterial stiffness between groups. Additionally, they found that HDL was significantly decreased in CVI patients.

Similarly, in terms of dyslipidemic condition, we showed that TG was significantly increased in CVI patients. It may be attributed to the roles of HDL and TG in chronic inflammation and atherosclerosis.

Weber T et al. investigated the relationship between left ventricular mass and brachial office as well as brachial and central ambulatory systolic blood pressure. They founded that in younger participants, central ambulatory systolic pressure was superior to both other measurements. Central ambulatory systolic pressure, measured with an oscillometric cuff, shows a strong trend toward a closer association with left ventricular mass and hypertrophy than brachial office/ambulatory systolic pressure [17]. In the present study, PWV and Aix values were higher in the CVI group compared to the control group, but however no significant differences were observed between the groups in terms of central systolic and diastolic blood pressures.

Association between symptoms of CVD in the lower extremities and cardiovascular risk factors was evaluated by Auzky O et al. and they showed that CVD symptoms were strongly associated with a higher prevalence of pathological values of ankle/brachial systolic blood pressure index (ABI) and several other manageable cardiovascular risk factors [18]. These findings support the data that CVD might also indicate increased risk for atherosclerosis. Therefore patients with CVI should be examined for cardiovascular diseases, even if they are apparently healthy subjects.

## 5. Conclusion

In conclusion, arterial stiffness is increased in patients with CVI. PWV and Aix are the most commonly used, easy, reproducible, reliable methods in the clinic to assess arterial stiffness. Logistic regression analysis showed that PWV and Aix were the independent predictors of CVI. PWV has the sensitivity of 88.9 % and specificity of 71.4 % to detect the presence of CVI.

## 6. Study Limitations

The main limitation of our study is a small patient population (110 patients) and cross-sectional study design. Since there is no follow-up in our study, it is not possible to determine the importance of this result in daily practice. Further studies are needed to determine the real diagnostic and prognostic role of these abnormalities in the clinical management of CVI patients.

## Figures and Tables

**Table 1:** Patient characteristics.

	All	Control group (n=48)	CVI group (n=62)	P Value
Age (±SD)	61.9±11.05	59.3±9.68	63.9±10.16	0.095

Female gender n(%)	60 (54.0)	28 (56.0)	40 (59.4)	0.072

BMI (kg/m2)	28.8±4.38	27.4±4.76	29.4±2.94	0.244

LVEF (%)	61.6±3.54	62.1±3.52	61.4±2.94	0.742

Hypertension n (%)	78 (68)	38 (69.0)	44 (72.2)	0.829

Diabetes mellitus n (%)	42 (40)	16 (29.8)	29 (47.2)	0.094

Hyperlipidemia n(%)	48 (41.7)	21 (44.0)	22 (36.1)	0.545

Smoking n(%)	68 (62.5)	30 (58.8)	45 (75.0)	0.096

Glucose (mg/dl)	94.56±10.04	92.42±8.9	95.12±9.5	0.395

HDL-C (mg/dl)	46.42±9.56	44.50±8.75	47.24±9.28	0.383

LDL-C (mg/dl)	123.85±35.83	123.67±33.30	123.97±37.65	0.967

TG (mg/dl)	132.65±55.86	122.50±45.65	158.74±58.72	0.040

Creatinine (mg/dl)	0.84±0.46	0.82±0.26	0.84±0.48	0.554

Uric acid (mg/dl)	5.36±1.30	5.31±1.22	5.39±1.35	0.706

SBP (mmHg)	123.1±10.2	122.6±9.97	123.5±10.52	0.657

DBP (mmHg)	78.4±8.2	77.7±8.90	79.0±8.13	0.423

Central SBP (mmHg)	118.4±8.1	116.9±9.45	119.3±9.32	0.262

Central DBP (mmHg)	81.6±9.3	80.7±8.92	83.0±8.36	0.159

PWV (m/sn)	8.56±1.62	8.03±1.43	8.92±1.65	0.001

Aix (%)	23.37±9.06	20.15±9.49	25.51±8.14	0.003

Independent T test (Bootstrap), Mann Whitney U test (Monte Carlo).

BMI: body mass index, LVEF: left ventricular ejection fraction, SD: standard deviation, HDL-C: high density lipoprotein cholesterol, LDL-C: low density lipoprotein cholesterol, TG: triglyceride, SBP: systolic blood pressure, DBP: diastolic blood pressure, PWV: pulse wave velocity, Aix: augmentation index.

**Table 2:** Correlation between arterial stiffness parameters and CVI.

	CVI	
	r	P
PWV	0.675	0.001

Aix	0.659	0.001

Partial Correlation Test,  r: Correlation coefficient,

PWV: pulse wave velocity, Aix: augmentation index,

CVI: chronic venous insufficiency.

**Table 3:** The independent predictors of chronic venous insufficiency in multivariate logistic regression analysis.

Variable	p	Odss Ratio (%95 C.I.)
PWV	<0.001	5.36 (2.35 – 12.26)

Aix	<0.001	4.69 (1.86 – 11.84)

TG	0.028	2.96 (1.34 – 6.55)

PWV: pulse wave velocity, Aix: augmentation index, TG: triglyceride, C.I.: confidence interval.

**Table 4:** Sensitivity and specificity.

	Cut-Off Value	AUC	Sensitivity (%)	Specificity (%)	p Value
PWV	9.2	0.826	88.9	71.4	<0.001

Aix	18.4	0.795	81.9	54.2	<0.001

ROC (Receiver Operating Curve) analysis (Honley & Mc Nell-Youden index J), AUC: area under the ROC curve, PWV: pulse wave velocity, and Aix: augmentation index.

## Data Availability

The data used to support the findings of this study are included within the article.

## References

[1] Bergan J. J., Schmid-Schönbein G. W., Smith P. D., Nicolaides A. N., Boisseau M. R., Eklof B. (2006). Chronic venous disease. *The New England Journal of Medicine*.

[2] Eberhardt R. T., Raffetto J. D. (2014). Chronic venous insufficiency. *Circulation*.

[3] Boutouyrie P., Tropeano A. I., Asmar R. (2002). Aortic stiffness is an independent predictor of primary coronary events in hypertensive patients: a longitudinal study. *Hypertension*.

[4] Willum-Hansen T., Staessen J. A., Torp-Pedersen C. (2006). Prognostic value of aortic pulse wave velocity as index of arterial stiffness in the general population. *Circulation*.

[5] Mitchell G. F., Hwang S. J., Vasan R. S. (2010). Arterial stiffness and cardiovascular events: the framingham heart study. *Circulation*.

[6] Maloberti A., Farina F., Carbonaro M. (2018). In healthy normotensive subjects age and blood pressure better predict subclinical vascular and cardiac organ damage than atherosclerosis biomarkers. *Blood Pressure*.

[7] Mitchell G. F., Moyé L. A., Braunwald E. (1997). Sphygmomanometrically determined pulse pressure is a powerful independent predictor of recurrent events after myocardial infarction in patients with impaired left ventricular function. SAVE investigators. Survival and ventricular enlargement. *Circulation*.

[8] Horinaka S., Yabe A., Yagi H. (2011). Cardio-ankle vascular index could reflect plaque burden in the coronary artery. *Angiology*.

[9] Laurent S., Boutouyrie P., Asmar R. (2001). Aortic stiffness is an independent predictor of all-cause and cardiovascular mortality in hypertensive patients. *Hypertension*.

[10] Mattace-Raso F. U. S., van der Cammen T. J. M., Hofman A. (2006). Arterial stiffness and risk of coronary heart disease and stroke: the Rotterdam Study. *Circulation*.

[11] Supiano M. A., Lovato L., Ambrosius W. T. (2018). Pulse wave velocity and central aortic pressure in systolic blood pressure intervention trial participants. *PLoS ONE*.

[12] Boutouyrie P., Bruno R. (2018). The Clinical Significance and Application of Vascular Stiffness Measurements. *American Journal of Hypertension*.

[13] Siriopol D., Covic A., Iliescu R. (2018). Arterial stiffness mediates the effect of salt intake on systolic blood pressure. *The Journal of Clinical Hypertension*.

[14] Mansilha A., Sousa J. (2018). Pathophysiological mechanisms of chronic venous disease and implications for venoactive drug therapy. *International Journal of Molecular Sciences*.

[15] Ozpelit E., Ozpelit M. E., Albayrak G. (2015). Arterial stiffness and cardiac functions in patients with chronic venous disease. *International Angiology: A Journal of the International Union of Angiology*.

[16] Aykan A. Ç., Menteşe S., Doğan E. (2016). Assessment of arterial stiffness in patients with chronic lower extremity venous disease: An observational study. *Phlebology*.

[17] Weber T., Wassertheurer S., Schmidt-Trucksäss A. (2017). Relationship between 24-hour ambulatory central systolic blood pressure and left ventricular mass: a prospective multicenter study. *Hypertension*.

[18] Auzky O., Lanska V., Pitha J., Roztocil K. (2011). Association between symptoms of chronic venous disease in the lower extremities and cardiovascular risk factors in middle-aged women. *International Angiology*.

